# Health Tract, No. 199

**Published:** 1865

**Authors:** 


HALL'S JOURNAL OF HEALTH. NOVEMBER. VOL. 12. No. 11. 1865.

### HEALTH TRACT, No. 199.

### STUDIO.

**WHERE TO STUDY.**—The air of a cellar is close, damp, musty, and vitiated; that of the house-top is clear, pure, and bracing. On the surface of the earth the atmosphere is cold, raw, and impure; on the mountains it is dry, rarified, and health-giving. The purer the air is, the more life does it impart to the blood, the more perfectly is the brain nourished, and the more vigorously does the mind work and the body move. Hence the "study" of the clergyman, the "office" of the physician and the lawyer, the "library" of the family, the "sitting-room" of the household, and the "chamber" of every sleeper, should always be in the upper stories, not merely for the greater purity of the air, but for a reason seldom thought of, and yet of very great sanitary value. The higher we ascend, the more rarified is the air, the greater bulk is required to impart a given amount of nourishment to the system; this greater rarity excites the instinct of our nature to deeper, fuller breathing, without any effort on our part, and this kind of breathing, as the reflecting must know, is antagonistic of consumption, that fell scourge of civilized society, which destroys full one sixth of the adult population. Hence the very suggestive remark of the distinguished naturalist Buffon: "All animals inhabiting high altitudes have larger lungs and more capacious chests than those which live in the valleys." In the same direction is the suggestive statement that in the city of Mexico, situated nine thousand feet above the level of the sea, of one hundred dying annually, only three are from consumption; while in our larger cities, but a few feet above the level of the sea, eighteen out of every hundred dead, perish from that disease. It should, therefore, be the aim of every student, of every sedentary person, of every invalid, to have the room in which a very large portion of the inactive part of life is spent, as far above the ground-floor as practicable, and in such a situation as will allow the sun to shine into it for the largest portion of each day, for this rarifies the air still more, and still more aids in developing and expanding the lungs by the greater depth and fullness of breathing which the increased atmospheric rarity induces.

